# Supplementary material for: Patterns of Intron Gain and Loss in Fungi
Source: PLoS Biol. 2004 Nov 30;2(12):e422. doi: 10.1371/journal.pbio.0020422 (PMC532390; doi:10.1371/journal.pbio.0020422)
Supplement: Table S1 — Also available at http://genes.mit.edu/NielsenEtAl/. (4.3 MB ZIP). [file pbio.0020422.st001.zip › NielsenEtAl/html/254.html]

AN6988.1.NCU05363.1.MG01380.2.FG01605.1


```
 CLUSTAL W (1.82) Multiple Sequence Alignments - Introns Inserted


Sequence 1: NCU05363.1	389 aa
Sequence 2: FG01605.1	389 aa
Sequence 3: MG01380.2	389 aa
Sequence 4: AN6988.1	389 aa
Alignment Length: 389 aa
Number Identitical Residues: 358 aa
Alignment Score (without introns) 13479


MG01380.2 	MALDNYYHNKIEAMKLEILKGQAVLRRLEAQRNDYNSRVRLLREELGLLQQPGSYVGEVV
NCU05363.1	MALDNYYHNKIEAMKLEILKGQAVLRRLEAQRNDYNSRVRLLREELGLLQQPGSYVGEVV
FG01605.1 	MALDEYYHNKIEAMKLEILKGQAALRRLEAQRNDYNSRVRLLREELGLLQQPGSYVGEVV
AN6988.1  	MALDNYYRNKIESMKLEIIQGQAVLRRLEAQRNDYNSRVRLLREELGLLQQPGSYVGEVV
          	****:**:****:*****::***.************************************

MG01380.2 	KVMGTKKVLVKVHPEGKYV1VDIADSVDITKLTVGKRVTLLSDSYKLEKMLPSSVDPLVS
NCU05363.1	KVMSTKKVLVKVHPEGKYV1VDVSDNVDITKLTVGKRVTLLSDSYKLEKMLPSSVDPLVS
FG01605.1 	KVMSTKKILVKVHPEGKYV1VDVSDSVDVAKLTPGKRVTLLSDSYKLEKMLPSSVDPLVS
AN6988.1  	KVMSTKKVLVKVHPEGKYV1VDIADGVDIGKLTVGKRVALLSDSYKLEKMLPSSVDPLVS
          	***.***:*********** **::*.**: *** ****:*********************

MG01380.2 	LMMVEKVPDSTYDMIGGLDQQIKEIKEVIELGLKHPELFESLGIAQPKGVLLYGPPGTGK
NCU05363.1	LMMVEKVPDSTYDMIGGLDQQIKEIKEVIELGLKHPELFESLGIAQPKGVLLYGPPGTGK
FG01605.1 	LMMVEKVPDSTYDMIGGLDQQIKEIKEVIELGLKHPELFESLGIAQPKGVLLYGPPGTGK
AN6988.1  	LMMVEKVPDSTYDMIGGLDQQIKEIKEVIELGLKHPELFESLGIAQPKGVLLYGPPGTGK
          	************************************************************

MG01380.2 	TLLARAVAHHTDCKFIRVSGSELVQKYIGEGSRMVRELFVMAREHAPSIIFMDEIDSIGS
NCU05363.1	TLLARAVAHHTDCKFIRVSGSELVQKYIGEGSRMVRELFVMAREHAPSIIFMDEIDSIGS
FG01605.1 	TLLARAVAHHTACKFIRVSGSELVQKYIGEGSRMVRELFVMAREHAPSIIFMDEIDSIGS
AN6988.1  	TLLARAVAHHTDCRFIRVSGSELVQKYIGEGSRMVRELFVMAREHAPSIIFMDEIDSIGS
          	*********** *:**********************************************

MG01380.2 	SRVEGTSGGDSEVQRTMLELLNQLDGFEPTKNIKVIMATNRLDILDPALLRPGRIDRKIE
NCU05363.1	SRVEGSSGGDSEVQRTMLELLNQLDGFEPTKNIKVIMATNRLDILDPALLRPGRIDRKIE
FG01605.1 	SRVEGSSGGDSEVQRTMLELLNQLDGFEPTKNIKVIMATNRLDILDPALLRPGRIDRKIE
AN6988.1  	SRIDSAGSGDSEVQRTMLELLNQLDGFEPTKNIKIIMATNRLDILDPALLRPGRIDRKIE
          	**::.:..**************************:*************************

MG01380.2 	FPPPS~VEARADILRIHSRKMNLTRGINLTKIAEKMNGCSGAELKGVCTEAGMYALRERR
NCU05363.1	FPPPS~VEARADILRIHSRKMNLTRGINLTKIAEKMNGCSGAELKGVCTEAGMYALRERR
FG01605.1 	FPPPS~VEARADILRIHSRKMNLTRGINLTKIAEKMNGCSGAELKGVCTEAGMYALRERR
AN6988.1  	FPPPS2VEARADILRIHSRSMNLTRGINLTKIAEKMNGCSGAELKGVCTEAGMYALRERR
          	***** *************.****************************************

MG01380.2 	VHVTQEDFELATAKILNKHDDKEV1SLGKLWK
NCU05363.1	VHVTQEDFELATAKILNKHDDKEV~SLAKLWR
FG01605.1 	VHVTQEDFELATAKILNKHDDKEV~SLGKLWK
AN6988.1  	VHVTQEDFDLATAKILNKHDDKEV~AVSKLFK
          	********:*************** ::.**::
```
